# Soccer health, performance, and injury prevention in highly trained youth soccer players: a public health approach

**DOI:** 10.3389/fpubh.2026.1937270

**Published:** 2026-09-14

**Authors:** Musa Lewis Mathunjwa, Seymur Aliyev

**Affiliations:** 1 Department of Human Movement Science, University of Zululand, KwaDlangezwa, South Africa; 2 Department of Coaching, Nakhchivan State University, Nakhchivan, Azerbaijan

**Keywords:** bio-banding, elite soccer, injury mitigation, load monitoring, physical performance, public health

## Abstract

**Background:**

The transition from youth academy participation to elite senior professional soccer is marred by physical volatility, non-linear development, and elevated injury risk, placing the long-term well-being of young athletes at the intersection of sports science and public health. Traditional chronological age group categorization exacerbates selection bias through the Relative Age Effect (RAE) and often fails to safeguard growing athletes from workload-induced injuries.

**Objective:**

This study aims to evaluate a multi-center, maturation-indexed public health intervention protocol designed to optimize athletic output, balance internal/external training loads, and systematically reduce non-contact lower-limb injuries in professional academy soccer players across early- to post-pubertal sub-groups (U13–U16).

**Methods:**

This protocol outlines a 24-week cluster-randomized controlled trial. A cohort of 240 elite male youth soccer players [categorized as Tier 3: Highly Trained/National Level per (1) aged 12–16 years] will be recruited from professional football academies (K = 4, *n* = 60 per cluster). Academies will be randomized in a 1:1 ratio to either standard chronological age-group care (Control) or a maturation-indexed framework (Experimental). The experimental arm integrates bio-banding protocols (grouping players by percentage of predicted adult height [%PAH]), progressive neuromuscular conditioning (FIFA 11+), and microtechnology-driven load threshold monitoring. Primary outcomes include injury incidence rate (per 1,000 h of exposure) and changes in physical performance profiles (sprint speed, repeated sprint ability, and vertical jump height). Secondary outcomes encompass internal-to-external load ratios and psychological wellness metrics (assessed via the Hooper index).

**Discussion:**

By treating elite youth soccer player development through a preventive public health lens, this trial will provide scalable evidence regarding the efficacy of biological maturation models. Aligning training load with physiological readiness aims to mitigate systemic youth injuries, reduce elite talent drop-out rates, and update international academy guidelines.

## Introduction

The systematic development of elite youth football players is a complex, multi-dimensional process that extends far beyond mechanical skill acquisition and short-term match performance. Modern professional soccer demands unprecedented physical thresholds, requiring young athletes to display high levels of linear speed, repeated sprint ability, tactical intelligence, and acute physiological resistance to injury (2, 3). As elite football academies globally integrate Long-Term Athlete Development (LTAD) frameworks into their pathways, sporting organizations encounter a critical performance–health paradox: the systematic drive to maximize athletic performance drastically intensifies the physiological load placed on adolescents, heavily escalating the clinical risk of overuse pathologies, soft-tissue tears, and growth-related conditions. At a physiological level, these overuse injuries in adolescent players are often heralded by specific micro-trauma markers, such as persistent serum creatine kinase elevation, localized apophyseal tenderness, and acute structural stiffness in muscle-tendon junctions.

Globally, epidemiological statistics reveal the severe burden of injuries within professional youth academies. Statistical data demonstrates that training and match injury incidence rates can fluctuate significantly depending on developmental phases, with a highly alarming surge occurring during adolescence (4, 5). However, regional and environmental variables across global academies—ranging from artificial vs. natural grass pitches to varying microcycle climate stressors—further modulate these epidemiological patterns. Specifically, proactive prevention protocols like the FIFA 11 + program highlight that non-contact anterior cruciate ligament (ACL) injuries alone pose a severe, career-threatening risk to youth players, though structured neuromuscular coordination models have shown the capacity to reduce these specific soft-tissue injuries by up to 45% demonstrate that structured neuromuscular coordination models can actively prevent and reduce acute non-contact lower-limb and anterior cruciate ligament (ACL) injuries by up to 45% in youth players (6, 7). Furthermore, during the critical transition into higher competitive brackets, adolescent football players encounter a substantial spike in high-intensity running volume. While these performance metrics are well-quantified, what remains clinically unknown is the precise, daily threshold where an individual’s external volume triggers a structural or musculoskeletal breakdown during periods of accelerated skeletal growth.

The core public health problem in youth football development stems from the traditional reliance on rigid chronological age brackets (e.g., U13, U14, U15) to organize squads and prescribe training volumes. Chronological age completely fails to capture the immense inter-individual variability observed during adolescence, specifically regarding the timing of puberty and the attainment of Peak Height Velocity (PHV) (8–10). The Relative Age Effect (RAE) represents a structural selection bias where athletes born earlier in the selection calendar year are disproportionately favored. In contrast, biological maturity timing (early, on-time, or late maturers) reflects an individual’s specific physiological rate of progress toward adult status, operating independently of birth month. Early-maturing players frequently exhibit temporary structural advantages in speed, power, and absolute strength, leading talent identification systems to disproportionately favor them. This creates a severe selection bias known as the Relative Age Effect (RAE), where late-maturing athletes with immense technical-tactical potential are systematically overlooked or dropped from elite pathways. Simultaneously, when uniform, age-prescribed workloads are applied blindly to a biologically diverse squad, growing players face mismatched physical strain, causing an alarming prevalence of growth-related injuries in players with rapid growth rates across pre-, circa-, and post-PHV periods (11).

A substantial knowledge gap persists because contemporary sports literature remains highly fragmented across isolated domains of maturation science, physical conditioning, and clinical sports medicine. Academy practitioners frequently utilize separate tools such as GPS-derived microtechnology tracking or biological somatic tracking (e.g., Khamis-Roche methods) without integrating them into a single, unified operational framework (12). Consequently, very few longitudinal studies have evaluated the protective public health efficacy of an integrated framework that dynamically modulates training stimuli based on real-time biological readiness.

To bridge this clear gap, this study protocol proposes a multi-center cluster-randomized trial aimed at evaluating an integrated, maturation-sensitive public health framework. The explicit aim of this trial is to determine whether combining somatic growth tracking (bio-banding) (13), progressive neuromuscular stabilization (FIFA 11+) (14), and microtechnology-driven load threshold monitoring (12) can significantly minimize non-contact lower-limb injury incidence rates while systematically optimizing the long-term physical performance profiles of elite academy soccer players. Crucially, this monitoring does not remain passive; somatic tracking and microtechnology data actively trigger individual workload caps and temporary reassignments to bio-banded groups when physical volatility or rapid growth spurts are detected. By shifting the development paradigm away from traditional, uniform chronological formatting and toward a fluid, biological-readiness framework, this study intends to provide scalable, empirical evidence to update international academy guidelines, safeguard adolescent welfare, and support sustainable talent development.

## Methods

### Study design and setting

This study is designed as a multi-center cluster-randomized trial based on the integration of Dynamic Systems Theory and the LTAD framework. The trial will be executed across professional academies following the Standard Protocol Items: Recommendations for Interventional Trials (SPIRIT) guidelines. The trial involves four academy clusters ( K=4 ), each providing 60 players across U13–U16 age categories ( n=60 per cluster). Assuming an Intraclass Correlation Coefficient (ICC) of ρ=0.03 for team-level injury rates, the design effect ( DE ) is:


DE=1+(m−1)ρ=1+(59×0.03)=2.77

For this protocol, LTAD is operationalised as a maturation-sensitive, stage-based development framework linking biological maturation, progressive physical development, training readiness, and injury prevention across the U13–U16 pathway. Recruitment will be conducted through four participating professional or semi-professional soccer academies, with each academy constituting one cluster and contributing approximately 60 male youth players (target total n = 240). The specific academy names/sites will be reported in the final protocol or trial record once institutional and partner permissions for disclosure have been confirmed.

Trial Registration: ClinicalTrials.gov NCT06425809 (Prospectively registered prior to participant enrollment).

### Participants and selection criteria

Elite youth football players enrolled in professional or semi-professional academies will be recruited. Athletes are categorized as Tier 3: Highly Trained/National Level per McKay et al. (1).

#### Inclusion criteria

- Youth players aged 12 to 16 years.
- Enrolled actively in a professional or semi-professional soccer academy.
- Signed parental/guardian informed consent and player assent.

#### Exclusion criteria

- Contraindications to high-intensity physical activity determined via standard pre-season cardiovascular screening.
- Pre-existing major injury causing extended training absence, defined as any time-loss musculoskeletal injury resulting in 28 or more consecutive days of absence from full training prior to baseline testing. Players with an active time-loss musculoskeletal injury who are unable to participate fully at baseline will be excluded until medically cleared for full participation.

Sample size and power analysis: Sample size was calculated using G∗Power for cluster-RCT designs. Based on published youth academy epidemiological data (baseline non-contact lower-limb injury rate of ~6.0 injuries per 1,000 exposure hours, SD=2.5 ), an alpha of 0.05, and 80% statistical power ( β=0.20 ) to detect a 30% reduction in lower-limb non-contact injury incidence, a minimum sample of 180 players is required. Accounting for an anticipated 25% attrition rate over 24 weeks due to transfers or dropouts, a total cohort of N=240 players across 4 participating academies (60 per academy) was targeted.

### Randomization and blinding

Following baseline screening (comprising somatic anthropometry, countermovement jump height, 10 m/30 m sprint timing, and 14-day baseline GPS/sRPE profiling), eligible academy clusters will be randomized into either the experimental or control sequence using a computer-generated randomization sequence performed by an independent statistician. Block randomization (block size of 2) stratified by academy facility tier will be used. Allocation concealment will be maintained using opaque, sequentially numbered sealed envelopes opened only after baseline testing. Due to the field-based nature of the intervention, coaches and players cannot be blinded to group assignment. However, outcome assessors and data analysts evaluating diagnostics will remain strictly blinded to the allocation groups to prevent detection and diagnostic bias. Unblinding for data analysts will occur only after the primary statistical models have been finalized and frozen.

### Interventions

**Table 1:** 

Component	Control group (standard care)	Experimental group (maturation-indexed intervention)
Squad organization	Grouped strictly by chronological birth year categories. Standard coach fidelity audits are logged fortnightly to ensure control protocols remain uninfluenced by bio-banding concepts.	Bio-Banded: Players will be grouped dynamically according to biological maturation using percentage of predicted adult height (%PAH) calculated with the Khamis-Roche method. Maturity status will be reassessed bi-monthly using standing height, seated height, body mass, and self-reported mid-parental height. Players will be assigned to pre-PHV, circa-PHV, or post-PHV bands according to the study’s pre-specified maturation classification. Where reassessment indicates a change in maturity classification, the player will be reassigned to the corresponding band before the subsequent bio-banded training block. Bio-banding will be implemented during 2 of 4 weekly training sessions, while chronological age-group organisation will be retained for weekend matches.
Neuromuscular warm-up	Standard coach-led physical warm-up or standard academy microcycle planning.	FIFA 11 + Program: Structured, evidence-based neuromuscular warm-up protocol executed prior to field training 3 times per week. Progression levels are applied every 6 weeks, validated by fortnightly coach fidelity audits.
Load management	Uniform squad running volumes and drill durations prescribed by chronological age group.	Individualized Load Monitoring: External and internal loads are strictly calibrated using GPS micro-technology, heart rate, and sRPE to prevent sudden spikes. Target Exponentially Weighted ACWR is maintained between 0.80 and 1.30. If ACWR exceeds 1.50 or weekly sRPE spikes by >15% , individual running volume is capped by 20–30% in the following microcycle. Players growing >0.6cm/month have high-speed running volume capped at 80% baseline.

### Data collection and outcome measures

#### Primary outcome measures

##### Physical performance evolution

- Speed and sprinting: Measured bi-monthly via linear timing gates (10 m and 30 m sprints) to track sprint adaptations.
- Power output: Assessed bi-monthly through vertical jump performance evolution using a force plate countermovement jump (CMJ) protocol.
- Injury incidence rate: Prospectively logged daily by club medical personnel following FIFA consensus definitions (15). For the primary outcome, a non-contact lower-limb injury will be classified as a time-loss injury when a physical complaint sustained during football training or match play prevents the player from taking full part in the next scheduled training session or match. Medical-attention injuries that do not result in time loss will be recorded separately as non-time-loss injuries to capture the broader injury burden. Injury severity will be recorded as the number of days unavailable for full participation. Injury diagnosis and mechanism will be coded using OSICS 10.1, and individual training and match exposure hours will be recorded to calculate injury incidence per 1,000 exposure hours.

##### Secondary outcome measures

- External workload parameters: Monitored continuously via 10 Hz GPS microtechnology units to log total distance, High-Speed Running (HSR), and acceleration/deceleration profiles.
- Internal load and stress: Calculated via session Rating of Perceived Exertion (sRPE, Borg CR-10 scale collected 30 min post-session) and heart rate parameters to identify cardiovascular strain and overtraining risks.
- Biological maturity status: Assessed non-invasively bi-monthly utilizing somatic tracking methods (Khamis-Roche method utilizing standing height, seated height, body mass, and self-reported mid-parental height) to determine proximity to Peak Height Velocity (PHV).
- Psychological wellness metrics: Assessed twice weekly prior to morning training using the Hooper Index (scoring sleep quality, fatigue, stress, and muscle soreness on a 1–7 scale).

### Data analysis

Data will be analyzed using mixed-effects linear regression models on an Intention-to-Treat (ITT) basis. Each group will be modeled as a fixed factor with random intercepts at both the academy cluster level and individual player level to adjust standard errors for clustering, adjusting for baseline covariates including baseline physical readiness, age, academy facilities/resources, and maturity status. Injury incidence rates will be calculated based on accumulated training and match exposure. Missing data resulting from player dropouts will be handled via multiple imputation by chained equations (MICE) under the missing-at-random assumption. Per-protocol sensitivity analyses will also be performed to evaluate the potential impact of protocol non-compliance and player dropouts on primary outcomes. Statistical significance is set at p<0.05 .

## Discussion

The findings of this anticipated multi-center cluster-randomized trial will provide critical, empirical evidence regarding the transition from rigid chronological age-group structures to fluid, maturation-indexed programming in elite youth football systems. Traditional chronological classification fails to capture the immense biological variation occurring during adolescence, which often results in severe selection bias, such as the Relative Age Effect (RAE) (16, 17). By systematically grouping players via bio-banding according to their percentage of predicted adult height, academies can mitigate mature-selection biases and ensure that late-maturing players are not systematically overlooked due to temporary physical performance deficits (16, 18–20).

A central strength of this protocol is its dual emphasis on performance optimization and systematic injury mitigation within a unified framework. The adolescent growth spurt and the period surrounding Peak Height Velocity (PHV) represent a phase of high physiological volatility, often characterized by temporary coordination deficits or “adolescent awkwardness” (21, 22). To manage this effectively, coaching staff will receive dedicated orientation modules to recognize transient movement regressions and differentiate true skill deterioration from temporary biological growth spurts. When uniform physical training loads are blindly applied to a biologically diverse squad, younger or late-maturing athletes frequently face mismatched physical strain, dramatically increasing the incidence of growth-related apophyseal conditions and non-contact soft-tissue injuries (23). Integrating the evidence-based FIFA 11 + neuromuscular warm-up program directly addresses this performance health paradox, targeting a significant reduction in lower-limb injuries, such as non-contact anterior cruciate ligament (ACL) tears (7, 24–26). In addition, FIFA 11 + programs have demonstrated effectiveness in reducing head and neck injuries, including concussion, among soccer players (27).

Furthermore, managing workloads using real-time microtechnology helps bridge a critical operational gap in academy player development. Combining external load parameters derived from GPS units with subjective internal stress indicators (such as session Rating of Perceived Exertion [sRPE]) provides practitioners with a comprehensive, individualized view of athlete fatigue and training adaptation (28–30). While GPS signal precision can experience slight variance in indoor facilities or densely urban covered pitches, supplementary inertial measurement units (IMUs) and localized positioning systems (LPS) will be utilized to ensure data continuity. This systematic monitoring serves as an early detection mechanism, safeguarding youth athletes from sudden spikes in training volume that are closely linked to overuse pathologies and soft-tissue breakdowns.

A potential limitation of this study protocol is its reliance on elite and semi-professional academy players within structured environments, which may limit the generalizability of the findings to grassroots or recreational youth setups where advanced monitoring technologies and sports science staff are unavailable. Additionally, the multi-center cluster design requires high compliance and coordination among field coaches, practitioners, and medical staff across consecutive microcycles (6, 31).

Ultimately, this study’s public health framework moves beyond immediate physical dominance, shifting the coaching and sports medicine focus toward long-term athlete protection, sustainable technical-tactical development, and structural welfare. By aligning training demands directly with an individual athlete’s evolving biological readiness, this model aims to provide health planners and national soccer associations with the necessary evidence to incorporate targeted, maturation-sensitive strategies into national elite developmental pathways.
